# Assessing the Impact of COVID-19 Vaccines on Sickle Cell Anaemia Patients: A Comparative Analysis of Biochemical and Haematological Parameters

**DOI:** 10.3390/biomedicines11082203

**Published:** 2023-08-05

**Authors:** Jehad A. Aldali, Badi A. Alotaibi, Hamzah J. Aldali, Glowi A. Alasiri, Ali Alaseem, Abdulaziz M. Almuqrin, Abdulrahman Alshalani, Fahad T. Alotaibi

**Affiliations:** 1 Department of Pathology, College of Medicine, Imam Mohammad Ibn Saud Islamic University (IMSIU), Riyadh 13317, Saudi Arabia; 2 Department of Clinical Laboratory Sciences, Collage of Applied Medical Sciences, King Saud Bin Abdulaziz University for Health Sciences, Riyadh 11481, Saudi Arabia; 3 King Abdullah International Medical Research Center, Riyadh 11481, Saudi Arabia; 4 Cellular and Molecular Medicine, College of Biomedical Science, University of Bristol, Bristol BS8 1QU, UK; 5 Department of Biochemistry, College of Medicine, Imam Mohammad Ibn Saud Islamic University (IMSIU), Riyadh 13317, Saudi Arabia; 6 Department of Pharmacology, College of Medicine, Imam Mohammad Ibn Saud Islamic University (IMSIU), Riyadh 13317, Saudi Arabia; 7 Chair of Medical and Molecular Genetics Research, Department of Clinical Laboratory Sciences, College of Applied Medical Sciences, King Saud University, Riyadh 12372, Saudi Arabia; 8 Department of Physiology, College of Medicine, Imam Mohammad Ibn Saud Islamic University (IMSIU), Riyadh 5701, Saudi Arabia

**Keywords:** COVID-19, vaccines, sickle cell anaemia, hematological parameters, biochemical parameters

## Abstract

The coronavirus disease 2019 (COVID-19) vaccines have been developed to help prevent the spread of the virus infections. The COVID-19 vaccines, including Pfizer, Moderna, and AstraZeneca, have undergone rigorous testing and have demonstrated both safety and effectiveness. Extensive evidence supports their effectiveness in preventing severe illness, hospitalization, and mortality associated with COVID-19 infection. The administration of COVID-19 vaccines can directly affect hematological and biochemical parameters, with reported cases showing an association with thrombosis and thrombocytopenia. Therefore, it was hypothesized that COVID-19 vaccines may also influence hematological and biochemical markers in sickle cell patients. This study aimed to investigate the side effects of COVID-19 vaccines on sickle cell patients, providing a comprehensive evaluation of hematological and biochemical parameters. To our knowledge, this is the first study of its kind conducted in Saudi Arabia. The study included the evaluation of Pfizer and Oxford-AstraZeneca vaccines in sickle cell patients, measuring key parameters. Our findings revealed varying impacts of both vaccines on the ALT, AST, and CRP levels. Notably, CRP and ALT exhibited potential as indicators for renal disease, diabetes, and arthritis. However, further investigations are necessary to uncover the underlying mechanisms that drive these observed differences and comprehend their clinical implications for this vulnerable patient population. The unique nature of our study fills a crucial research gap and underscores the need for additional research in this area.

## 1. Introduction

The emergence of the coronavirus disease 2019 (COVID-19) pandemic caused by the SARS-CoV-2 has presented an unprecedented global health challenge. The coronavirus family, which descended from zoonotic events, has become a prevalent family of human pathogenic viruses over the past two decades. SARS-CoV first appeared in China in 2003, where it caused 8422 infectious cases and 916 fatalities in 26 different countries [1]. Similarly, the MERS-CoV coronavirus spread in Saudi Arabia and other Middle Eastern countries in 2012, resulting in more than 2949 infectious cases and 858 deaths [1]. COVID-19 infection displays a wide range of symptoms and outcomes, with certain factors such as age, comorbidity, co-infection, and compromised immune systems contributing to a higher risk of severe illness. Since December 2020, several variants of concern with higher transmissibility or the ability to evade prior immunization have been reported [2,3,4].

Since the beginning of the COVID-19 pandemic, efforts have been made to reduce the risk of infection transmission in hospitals and the community [5]. Several vaccines have been developed and proven to be effective [1,6]. The World Health Organization authorized a few vaccinations for emergency use in early 2021 [7,8]. ChAdOx1 nCoV-19 vaccine (also known as Oxford AstraZeneca) and the BNT162b2 vaccine (also known as Pfizer) were the first two vaccines introduced and licensed in the Kingdom of Saudi Arabia. [9]. The COVID-19 vaccinations were associated with a number of mild to moderate side effects, including headaches, discomfort, edema, and redness at the injection site, as well as muscle and joint issues [10,11].

COVID-19 has been associated with various hematological complications such as anemia, thrombocytopenia, and disseminated intravascular coagulation [12,13,14]. In the context of COVID-19, individuals with sickle cell disease (SCD) are more vulnerable to experiencing severe disease compared to the general population [15]. Indeed, viral infections can cause a vaso-occlusive crisis (VOC) and necessitate hospitalization in some cases [15]. Further, SARS-CoV-2 tropism in lung tissues and the increased risk of pulmonary embolism induced by this virus raise concerns in SCD patients, for whom acute chest syndrome (ACS) is a primary cause of early mortality. Apart from specific COVID-19-related illnesses, some viral infections in patients with SCD could lead to painful obstructive crises and life-threatening acute chest syndromes. COVID-19 could therefore have devastating consequences for these patients [16].

SCD is an inherited red blood cell illness caused by a single amino acid mutation in the human hemoglobin protein’s beta chain [17,18]. Chronic hemolytic anemia, repeated vascular occlusion, insidious vital organ deterioration, early mortality, and poor quality of life are all symptoms of SCD. In addition, repeated vaso-occlusive episodes in childhood can cause functional asplenia and immunological insufficiency, leading to a life-long increased vulnerability to deadly bacterial infections. Acute and chronic pain, as well as other significant health issues such as acute chest syndrome (ACS) and stroke, is common in SCD patients [19,20]. SCD patients are at an increased risk of developing severe complications from COVID-19 infection due to their weakened immune system and underlying health conditions [21]. Recent studies have shown that individuals with SCD who contract COVID-19 are more likely to experience severe symptoms, require hospitalization, and they are at a higher mortality risk than the general population [22,23]. Previous studies have shown that adults with SCD usually have mild to moderate COVID-19 disease but are at higher risk of COVID-19-related hospitalization and death compared to adults without SCD [21,24,25]. Most children with SCD and COVID-19 infection encounter mild illness; however, those with sickle cell anemia (SCA)-related comorbidities are more likely to require hospitalization [26]. Nonetheless, compared to adults with or without SCD, children with SCA have lower chances of experiencing severe sickness or death related to COVID-19 [21].

Studies showed that there is some degree of hesitation among SCD patients in receiving COVID-19 vaccines [27]. Furthermore, given the recognized link between inflammation and sickle cell crisis, there is fear that an immunological response to vaccination may cause a vaso-occlusive crisis or a considerable increase in the known adverse effects of vaccination [28] COVID-19 vaccines such as Pfizer, Moderna, J&J, and AstraZeneca have undergone extensive testing and have been found to be safe and effective in preventing COVID-19-related severe illness, hospitalization, and death in the general population [29,30]. Therefore, the Centers for Disease Control and Prevention (CDC) have designated individuals with SCD as a population that requires COVID-19 vaccination. 

Multiple studies reported mild to severe adverse effects in healthy COVID-19 vaccine recipients. Some serious side effects include neurological complications such as transverse myelitis and, more importantly, blood clotting disorders such as thrombocytopenia and thrombosis. These cases were mostly reported in people younger than 50 years old within 1–3 weeks after receiving one of the COVID-19 vaccine candidates, ChAdOx1 nCoV-19 [31,32]. Three SCD patients were reported to have experienced VOC within six days of receiving the ChAdOx1 nCov-195-7/AstraZeneca vaccine, according to a recent publication [33]. This finding raises questions about the safety of COVID-19 vaccinations for this population. To address this gap in knowledge, we sought to assess the impact of COVID-19 vaccines on SCA patients with regard to the hematological and biochemical parameters. Our study is the first to shed light upon hematological biochemical parameters in response to different types of COVID-19 vaccinations in SCA patients. The combination of hematological and biochemical parameters can provide an accurate analysis of the side effects of COVID-19 vaccines on the SCA population. 

## 2. Method

### 2.1. Study Design and Settings 

The present retrospective research was conducted between April 2020 and February 2022 at the King Abdullah International Medical Research Centre (KAIMRC), Ministry of National Guard, Health Affairs, in Riyadh, Saudi Arabia. This study included sickle cell anemia (SCA), either homozygous or heterozygous patients, who received COVID-19 vaccines (Oxford-AstraZeneca vaccine or Pfizer-BioNTech vaccine). Patients were divided into two groups; SCA patients who received the Oxford-AstraZeneca vaccine (AstraZeneca Group, Cambridge, UK) and SCA patients who received the Pfizer-BioNTech vaccine (Pfizer Group, New York, NY, USA).

### 2.2. Data Collection 

After receiving approval from the ethics committee, members of the research team got in touch with the medical records unit, department of research, and data management section at the King Abdullah International Medical Research Centre (KAIMRC), which is located in the Ministry of National Guard-Health Affairs in Riyadh, Saudi Arabia. This was conducted for the purpose of collecting data. The information was gathered from the patients’ electronic health records using the data management tools that were present in the hospital. During the study period, the data of a total of 159 sickle cell, either homozygous or heterozygous, patients who received doses of the COVID-19 vaccines were collected. The laboratory data were collected after the last dose. Most blood samples were collected within 12–16 months post-vaccinations. The research team also analyzed various diseases in SCA patients such as arthritis, cancer, depression, diabetes, hyperlipidemia, and hypertension. Biochemical parameters of SCA patients (pre-COVID-19 dose and post-COVID-19 dose) including alanine transferase (ALT), aspartate aminotransferase (AST), creatinine, and C-reactive protein (CRP) were collected and analyzed. Also, hematological parameters (pre-COVID-19 dose and post-COVID-19 dose) including white blood cell count (WBC), platelet count (Plt), hemoglobin (Hgb), iron, and ferritin were collected and analyzed.

### 2.3. Statistical Analysis 

Statistical analyses were performed using SPSS^®^ software version 25.00 (IBM Corp., Armonk, NY, USA), and graphical representation was generated using GraphPad Prism version 9.4.1 (GraphPad Software Inc., San Diego, CA, USA). The normality of the data was determined with the Shapiro–Wilk test. Baseline data were summarized using medians and interquartile ranges (IQR) for continuous data or percentages and frequencies for categorical data. Baseline differences between study groups were assessed by a Mann–Whitney test for continuous data and Pearson’s chi-square test for categorical data. For the biochemical and hematological parameters, a repeated t-test was performed to evaluate pre-vaccine and post-vaccine differences between the two groups if the data were normally distributed. If the normality assumption was not obtained, the Wilcoxon test was performed. A *p*-value of less than 0.05 was considered significant. If the statistical test revealed significant differences between pre-vaccine and post-vaccine in any of the biochemical and hematological parameters, chi-square tests were followed to test the association between the significant differences in the biochemical and hematological parameters and the baseline variables. 

## 3. Results

### 3.1. Study Population

The total number of SCD patients included in the study was 159. The group who received the Oxford-AstraZeneca vaccine consisted of 94 patients, and 65 patients received the Pfizer-BioNTech vaccine. The hematological and biochemical parameters were analyzed after the last vaccine dose, within a few weeks to 18 months post-vaccination. Both groups in this study did not present a significant difference for the baseline characteristics (Table 1). Approximately, half of the participants were females (*n* = 45; 47.9% in the AstraZeneca group and *n* = 36; 55.4% in the Pfizer group). The average age was 29 years (23–38.25) in the AstraZeneca group, and 27 years (19–35) in the Pfizer group. The numbers of patients who showed post-vaccination side effects were only 5 (6.5%) and 9 (8.5%) for AstraZeneca and Pfizer groups, respectively.

### 3.2. Biochemical and Hematological Parameters

**Figure 1:**
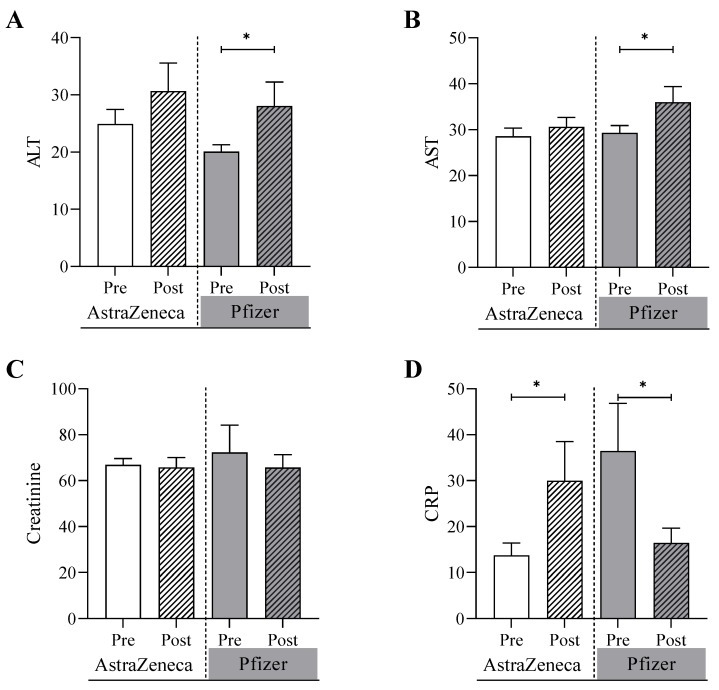
Biochemical analyses of (**A**) alanine transferase, ALT, (**B**) aspartate aminotransferase, AST, (**C**) creatinine, and (**D**) C-reactive protein, CRP, conducted on the AstraZeneca group and the Pfizer group before and after receiving one of the COVID-19 vaccines. * Denotes a significant difference (* *p* < 0.05).

**Figure 2:**
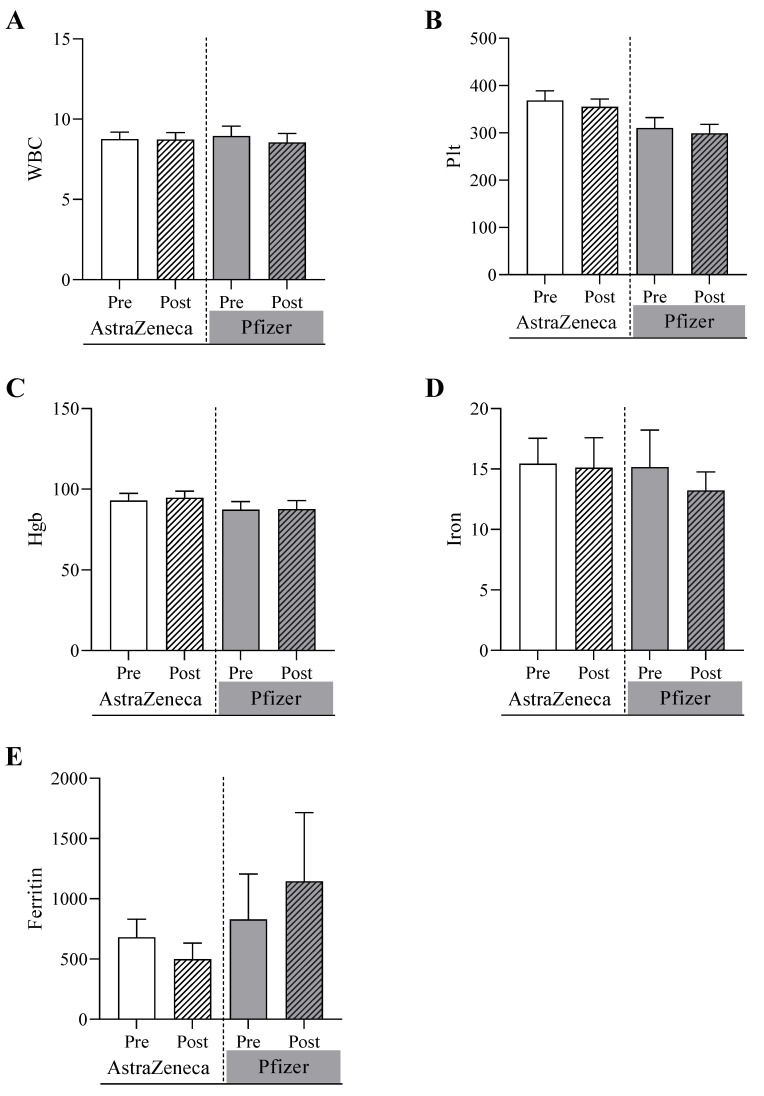
Hematological analyses for pre- and post-AstraZeneca and Pfizer vaccinations of (**A**) white blood cell count, WBC, (**B**) platelet count, Plt, (**C**) hemoglobin, Hgb, (**D**) iron, and (**E**) ferritin conducted on the AstraZeneca group and the Pfizer group before and after receiving of the COVID-19 vaccines.

The biochemical tests were conducted for both the AstraZeneca group and Pfizer group before and after receiving doses of the COVID-19 vaccines (Figure 1). It was shown that alanine transferase (ALT) was significantly increased in the Pfizer group from 20.1 U/L (SEM = 1.1) at the pre-vaccine testing point to 28.1 U/L post-vaccine testing point (*p* value = 0.04). In contrast, ALT increased, but not significantly, in the AstraZeneca group from 24.9 U/L (SEM = 2.2) at pre-vaccine testing point to 30.7 U/L (3.9) at the post-vaccine testing point (*p* value = 0.20). Similarly, the concentration of aspartate aminotransferase (AST) was significant, increasing in the Pfizer group from 29.3 U/L (1.5) to 36.0 (3.0) (*p* value = 0.05) while the concentration was marginally changed from 28.6 U/L (1.6) to 30.6 U/L (1.7) (*p* value = 0.69) in the AstraZeneca group at pre-vaccine and post-vaccine testing points. The C-reactive protein (CRP) level was significantly increased in the AstraZeneca group from 13.7 mg/L at pre-vaccine testing point to 30.0 mg/L at the post-vaccine testing point (*p* value = 0.01). In contrast, the CRP level was significantly decreased in the Pfizer group in the post-vaccine testing point (*p* value = 0.05). No significant differences were detected between the two study groups in the creatinine levels and the hematological parameters including WBC count, platelets, hemoglobin, iron, and ferritin levels (Figure 2). To analyze the correlation between age, BMI, and laboratory test values after doses of the COVID-19 vaccines in SCA patients, the following results were obtained in Table 2. There were significant association between sex and renal disease with CRP. Also, ALT values are significantly associated with diabetes and arthritis patients. 

## 4. Discussion

It has been well established that individuals with hemoglobinopathies might be at higher risk of severe illness if they contract COVID-19. On the other hand, COVID-19 vaccines have undergone extensive clinical trials to ensure their safety and efficacy, as well as the clinical and biochemical markers’ implications following the COVID-19 vaccination. Therefore, to attain a high level of public health, COVID-19 vaccines have been recommended for most individuals, including those with underlying health conditions such as sickle cell anemia. The aim of this study was to elucidate the biochemical markers of pre- and post-COVID-19 vaccination with both AstraZeneca and Pfizer within SCD patients.

The immunological markers were found to be significantly affected following vaccination within SCD patients. A prior national report has assessed the diverse impacts of COVID-19 vaccines on individuals with hemoglobinopathies. The study assessed the immune response to COVID-19 vaccines in patients with hemoglobinopathies, including SCD, by measuring both binding and neutralizing antibodies (nAbs). However, the study did not include an assessment of the safety profile of this particular population. Understanding the safety of the COVID-19 vaccination in this specific group is equally important to ensure the overall well-being and efficacy of vaccination efforts. Recently, there have been several reports of unexpected thrombotic events and thrombocytopenia after administration of different COVID-19 vaccines [34,35]. Similar events were previously recorded in children after receiving the measles, mumps, and rubella (MMR) vaccine [36]. Although such incidents are uncommon, the public may be reluctant to be vaccinated because of the perceived risk of severe side effects [36]. 

The Pfizer-BioNTech vaccine is based on a modified mRNA molecule that encodes for the spike (S) protein of SARS-CoV-2. On the other hand, the Oxford-AstraZeneca vaccine is based on a replication-deficient chimpanzee adenoviral vector that encodes for the SARS-CoV-2 S protein. Both vaccines aim to generate a sufficient titer of S antibodies, thereby preventing virus entry into host cells [37]. The study included a total of 159 patients, with 94 patients receiving the Oxford-AstraZeneca vaccine and 65 patients receiving the Pfizer-BioNTech vaccine. The groups did not differ significantly in terms of baseline characteristics, ensuring a comparable study population. Regarding the biochemical tests conducted on the patients, the concentration of alanine transferase (ALT) was significantly increased in the Pfizer group following vaccination, whereas the increase in the AstraZeneca group was not statistically significant. The exact reason for the ALT increase in the vaccinated individuals is not fully understood; however, the elevated levels suggest that there is an association between the Pfizer vaccine and liver oxidative stress and inflammation in SCD patients. Vaccination may cause a protective immune response, which causes temporary liver inflammation and increased ALT production. In addition, external factors may contribute to elevated ALT levels, including medications, alcohol consumption, or infections. Interestingly, the elevated levels of ALT in SCD patients were found high in the post-Pfizer-BioNTech vaccinated group, but not in Oxford-AstraZeneca vaccinated group.

Interestingly, C-reactive protein (CRP), a liver-producing inflammatory marker, exhibited a contrasting pattern between the two vaccine groups, indicating a clear inflammatory response. In this study, we examined a comparative analysis of the effect of these vaccines on C-reactive protein (CRP) levels. The comparative analysis of CRP level variations in the AstraZeneca and Pfizer vaccine groups provides valuable insight into the immune responses elicited by these vaccines. The observed increase in CRP levels in the AstraZeneca group, post-vaccination, and the decrease in CRP levels in the Pfizer group, post-vaccination, demonstrate the need for continued research into the underlying mechanisms responsible for these differences. It was previously found that COVID-19 vaccination from both AstraZeneca and Pfizer can elevate CRP levels [38,39]. However, we found that the level of CRP significantly decreased following the Pfizer vaccination. It was thought that comorbidity and/or coinfection may be the cause of the elevated CRP levels prior to Pfizer vaccination. Therefore, the recovery following the vaccination did not lead to a continuous high level of CRP. In addition, multiple variables may account for the disparities in CRP levels between the AstraZeneca and Pfizer groups. First, it is crucial to recognize that AstraZeneca and Pfizer vaccines utilize distinct vaccine technologies. AstraZeneca uses a platform based on adenoviral vectors, while Pfizer employs mRNA technology. These disparities in vaccine platforms may result in varying immune responses and inflammation patterns, which may explain the variances in CRP levels. 

Regarding the post-vaccination side effects, the study reported a slightly higher proportion of patients experiencing side effects in the Pfizer group compared to the AstraZeneca group. However, the differences were not statistically significant. While it is important to monitor and manage any adverse events following vaccination, the absence of significant differences between the two groups indicates a comparable safety profile for both vaccines in SCD patients. In contrast, the hematological analysis showed no significant differences detected between the two vaccination groups. The parameters examined included white blood cell count, platelet count, hemoglobin, iron, and ferritin. These findings suggest that both Pfizer and AstraZeneca vaccines have negligible effects on hematological parameters in SCD patients. A previous study has assessed the hematological parameters and thrombotic profiles of healthy individuals who received the Pfizer-BioNTech (BNT162b2) COVID-19 vaccine [40]. The study also did not find any significant changes in the hematological parameters between the time before vaccination (day 0) and 14 to 21 days after vaccination. Additionally, all participants had negative anti-PF4 antibodies following vaccination. These findings suggest that the incidence of hematological abnormalities or induction of anti-PF4 antibodies following Pfizer-BioNTech (BNT162b2) vaccination were highly unlikely. However, the sample size of that study was relatively small, including only 38 patients. Therefore, studies with larger sample sizes evaluating hematological parameters at different time points following vaccination might confidently confirm the absence of hematological parameter changes following COVID-19 injection. Our study provides a more comprehensive overview of the observed association by spotting a larger cohort and studying a larger number of parameters, focusing on the SCD patients in Saudi Arabia.

Our study also explored the correlation between significant differences in biochemical parameters and baseline variables. Our results revealed significant differences in the CRP values in renal diseases and in gender. CRP is considered a risk marker for the loss of renal function. The suggested mechanism indicates that early inflammatory processes associated with high body fat may lead to glomerular hyperfiltration rate-induced loss of renal function [40]. Khera et al., (2009) concluded that CRP levels increase with changes in fat mass and are more affected by fat distribution in females. Obesity as a trigger of subclinical inflammation may be of relevance to females more than males [41]. 

A retrospective study was conducted to study CRP as a surrogate marker to identify the proportion of patients with inflammatory arthritis who had a flare of their rheumatological condition within 4 weeks of receiving a coronavirus disease 2019 (COVID-19) vaccine [42]. The study included 1620 adults (mean age 61 years, 64% female). AstraZeneca (AZ), BioNTech-Pfizer, and Moderna vaccines were administered. Vaccine uptake was 95.2% for the first dosage (1542 of 1620), 95.7% for the second dose (1550 of 1620), and 88.7% for the third dose (1437 of 1620). Within 30 days following their immunization, 192 of 1542 patients (12.5%) had a CRP increase of >10 mg/L, which was higher than the baseline flare rate of 8.6%. 

Although the findings demonstrate a statistically significant increase in illness flare (12.5% vs. a baseline rate of 8.6%), the clinical relevance of this increase is uncertain. SARS-CoV-2 infection, on the other hand, has been demonstrated numerous times to be an independent risk factor for rheumatic illness flare, ranging from 20–40% [43,44,45]. As a result, people with inflammatory arthritis should still be advised to be vaccinated against COVID-19. According to this study, there is an increase in CRP following COVID-19 immunization in patients with inflammatory arthritis, which may indicate that it can cause a disease flare. Additional studies are needed to better inform and counsel patients since ongoing booster vaccinations are scheduled for rheumatology patients. Furthermore, this study emphasizes the importance of closely monitoring patients with inflammatory arthritis for disease flare-ups and prompt intervention to avoid loss of disease control.

There is another finding that might show a possible interpretation about the effect of the COVID-19 vaccines on CRP. La Gualana et al., (2023) revealed that except for the lipid carrier, which contains lipid nanoparticles rather than lipoplex, the Pfizer/BioNTech BNT162b2 mRNA vaccine against SARS-CoV-2 is identical to a noninflammatory tolerogenic mRNA vaccine (MOGm1Ψ). The results show that immunization with BNT162b2 increased the frequency and absolute count of CD4^pos^CD25^high^CD127^low^ putative Treg cells, whereas vaccination with the adenovirus-vectored ChAdOx1 nCoV-19 vaccine decreased the frequency and absolute count of CD4^pos^CD25^high^CD127^low^ cells, suggesting that ChAdOx1 nCoV-19 is more effective at priming T cell immunity as well as mRNA and adenovirus-vectored vaccines have intrinsic immunomodulatory features in addition to the potential to produce antigen-specific responses [42].

In addition, ALT values are significantly upregulated in diabetes and arthritis patients. Saligram et al., (2012) showed a high frequency of elevated ALT levels in newly diagnosed patients with Type 2 diabetes (T2DM), suggesting that developing liver abnormalities associated with dysglycemia might be predisposing factors for developing T2DM. Abnormal ALT levels are associated with features of metabolic syndrome [46]. Fahmy et al., (2018), reported cases of liver function test abnormalities, including ALT levels, in patients with inflammatory arthritis. The study showed that abnormal liver function tests are common in patients with inflammatory arthritis and are often caused by disease-modifying anti-rheumatic drugs [47]. These results indicate that ALT might be indicative of diabetes and arthritis diseases. However, this aspect of the analysis was not specifically addressed in the information provided in our study. To comprehensively assess the relationship between biochemical changes and baseline characteristics, further statistical analyses, such as regression or correlation analysis, are necessary. 

## 5. Limitations

The present investigation is subject to many limitations. Firstly, it is worth noting that this study encompassed all individuals diagnosed with sickle cell disease, irrespective of their specific subtypes, such as homozygous or heterozygous cases. The present investigation does not allow for the determination of whether the observed findings are attributable to the specific subtype of sickle cell syndromes or the type of COVID-19 vaccinations. Additionally, this presents the challenge of subject variability, which might impact the uniformity within groups and hence reduce the statistical power to identify any disparities across the groups. However, Table 1 illustrates that there are no statistically significant differences observed between the groups, suggesting that the subjects included in the study were randomly selected and evenly distributed across the two groups. 

One further constraint of the present investigation is its observational design. Hence, it is plausible that certain external variables, not accounted for in our investigation, could potentially introduce confounding factors that may impact the statistical analysis. One instance of these confounding factors is the quantity of transfusions administered to patients, which has the potential to influence the outcomes. Another aspect pertaining to this observational study is its lack of a biological rationale for the observed results, which might be attributed to its exploratory design. Regrettably, the study lacked the inclusion of supplementary parameters that could potentially elucidate the notable alterations observed in the bio-chemical parameters, specifically pertaining to the levels of inflammatory cytokines and iron metabolism parameters. Further research is required to substantiate these findings and have a comprehensive understanding of the ramifications of COVID-19 vaccines on individuals with sickle cell anemia.

## 6. Conclusions

This study provided insights into the biochemical and hematological changes in SCD patients following the COVID-19 vaccines Oxford-AstraZeneca and Pfizer-BioNTech. The findings suggested that the two vaccines may have varying effects on certain biochemical parameters, such as ALT, AST, and CRP while displaying comparable impacts on hematological parameters. Also, our study concluded that CRP and ALT might be used as indicators for renal diseases, diabetes, and arthritis diseases. These results emphasize the importance of considering the specific vaccine used when evaluating potential effects on patients with underlying conditions such as sickle cell anemia. Further research is needed to elucidate the mechanisms underlying these observed differences and their clinical implications for this vulnerable patient population.

## Figures and Tables

**Table 1:** Baseline characteristics of sickle cell anemia (SCA) patients included in the study.

Variables	AstraZeneca Group (*n* = 94)	Pfizer Group (*n* = 65)	*p-*Value
**Demographics**			
Sex (female), *n* (%)	45 (47.9)	36 (55.4)	0.36
Age (years), median (IQR)	29 (23–38.25)	27 (19–35)	0.21
BMI (kg/m^2^), median (IQR)	25.3 (22.7–28.9)	24.0 (21.0–28.9)	0.33
**Chronic comorbidity**			
Arthritis, *n* (%)	12 (12.8)	3 (4.6)	0.09
Asthma, *n* (%)	1 (1.1)	2 (3.1)	0.36
Cancer, *n* (%)	3 (3.2)	1 (1.5)	0.51
Depression, *n* (%)	7 (7.4)	4 (6.2)	0.75
Diabetes, *n* (%)	29 (30.9)	14 (21.5)	0.20
Heart failure, *n* (%)	0 (0)	1 (1.5)	0.23
Hyperlipidemia, *n* (%)	5 (5.3)	3 (4.6)	0.84
Hypertension, *n* (%)	7 (7.4)	3 (4.6)	0.47
Renal disease, *n* (%)	5 (5.3)	1 (1.5)	0.22
Medication, *n* (%)	66 (71)	53 (81.5)	0.13
**Vaccine-related parameters**			
Post-vaccination side effects, *n* (%)	5 (6.5)	9 (8.5)	0.43

BMI, Body mass index.

**Table 2:** Partial correlation analyses between the differences in the biochemical parameters and the baseline variables.

Variables		ALT	AST	CRP
Sex	Correlation Coefficient	−0.127	−0.107	−0.259
	*p*-value	0.21	0.26	0.03 *
Age	Correlation Coefficient	−0.101	0.119	0.021
	*p*-value	0.31	0.21	0.87
BMI	Correlation Coefficient	0.125	−0.140	0.124
	*p*-value	0.20	0.14	0.33
Arthritis	Correlation Coefficient	0.187	0.082	0.101
	*p*-value	0.04 *	0.38	0.42
Asthma	Correlation Coefficient	−0.032	0.023	−0.095
	*p*-value	0.74	0.81	0.45
Cancer	Correlation Coefficient	−0.125	0.103	0.008
	*p*-value	0.20	0.27	0.92
Depression	Correlation Coefficient	−0.036	0.032	−0.102
	*p*-value	0.71	0.74	0.42
Diabetes	Correlation Coefficient	0.185	−0.034	0.115
	*p*-value	0.04 *	0.72	0.36
Heart failure	Correlation Coefficient	−0.036	−0.009	0.033
	*p*-value	0.71	0.92	0.86
Hyperlipidemia	Correlation Coefficient	−0.101	0.098	0.063
	*p*-value	0.30	0.30	0.62
Hypertension	Correlation Coefficient	−0.115	0.050	0.063
	*p*-value	0.24	0.60	0.62
Renal disease	Correlation Coefficient	−0.099	0.101	0.277
	*p*-value	0.31	0.28	0.03 *
Medication	Correlation Coefficient	0.128	−0.111	−0.044
	*p*-value	0.19	0.24	0.73
Post- vaccination side effects	Correlation Coefficient	0.166	−0.145	−0.211
	*p*-value	0.09	0.08	0.09

ALT, alanine transferase; AST, aspartate aminotransferase; CRP, C-reactive protein; BMI, body mass index. * Denotes a significant difference (* *p* < 0.05).

## Data Availability

The data are available upon request from the corresponding author.

## References

[1] Sharma A., Tiwari S., Deb M.K., Marty J.L. (2020). Severe acute respiratory syndrome coronavirus-2 (SARS-CoV-2): A global pandemic and treatment strategies. Int. J. Antimicrob. Agents.

[2] Kidd M., Richter A., Best A., Cumley N., Mirza J., Percival B., Mayhew M., Megram O., Ashford F., White T. (2021). S-variant SARS-CoV-2 lineage B1. 1.7 is associated with significantly higher viral load in samples tested by TaqPath polymerase chain reaction. J. Infect. Dis..

[3] Volz E., Mishra S., Chand M., Barrett J.C., Johnson R., Geidelberg L., Hinsley W.R., Laydon D.J., Dabrera G., O’Toole Á. (2021). Assessing transmissibility of SARS-CoV-2 lineage B. 1.1. 7 in England. Nature.

[4] Sheikh A., McMenamin J., Taylor B., Robertson C. (2021). SARS-CoV-2 Delta VOC in Scotland: Demographics, risk of hospital admission, and vaccine effectiveness. Lancet.

[5] Shrotri M., Swinnen T., Kampmann B., Parker E.P. (2021). An interactive website tracking COVID-19 vaccine development. Lancet Glob. Health.

[6] Althobaity Y., Wu J., Tildesley M.J. (2022). Non-pharmaceutical interventions and their relevance in the COVID-19 vaccine rollout in Saudi Arabia and Arab Gulf countries. Infect. Dis. Model..

[7] Dal-Ré R., Caplan A.L., Gluud C., Porcher R. (2021). Ethical and scientific considerations regarding the early approval and deployment of a COVID-19 vaccine. Am. Coll. Physicians.

[8] Tumban E. (2020). Lead SARS-CoV-2 candidate vaccines: Expectations from phase III trials and recommendations post-vaccine approval. Viruses.

[9] Wiersinga W.J., Rhodes A., Cheng A.C., Peacock S.J., Prescott H.C. (2020). Pathophysiology, transmission, diagnosis, and treatment of coronavirus disease 2019 (COVID-19): A review. JAMA.

[10] Polack F.P., Thomas S.J., Kitchin N., Absalon J., Gurtman A., Lockhart S., Perez J.L., Pérez Marc G., Moreira E.D., Zerbini C. (2020). Safety and efficacy of the BNT162b2 mRNA Covid-19 vaccine. N. Engl. J. Med..

[11] Voysey M., Clemens S.A.C., Madhi S.A., Weckx L.Y., Folegatti P.M., Aley P.K., Angus B., Baillie V.L., Barnabas S.L., Bhorat Q.E. (2021). Safety and efficacy of the ChAdOx1 nCoV-19 vaccine (AZD1222) against SARS-CoV-2: An interim analysis of four randomised controlled trials in Brazil, South Africa, and the UK. Lancet.

[12] Hariyanto T.I., Kurniawan A. (2020). Anemia is associated with severe coronavirus disease 2019 (COVID-19) infection. Transfus. Apher. Sci..

[13] Rahi M.S., Jindal V., Reyes S.-P., Gunasekaran K., Gupta R., Jaiyesimi I. (2021). Hematologic disorders associated with COVID-19: A review. Ann. Hematol..

[14] Castro R.A., Frishman W.H. (2021). Thrombotic complications of COVID-19 infection: A review. Cardiol. Rev..

[15] Arlet J.B., Lionnet F., Khimoud D., Joseph L., de Montalembert M., Morisset S., Garou A., Cannas G., Cougoul P., Guitton C. (2022). Risk factors for severe COVID-19 in hospitalized sickle cell disease patients: A study of 319 patients in France. Am. J. Hematol..

[16] Arlet J., de Luna G., Khimoud D., Odièvre M., de Montalembert M., Joseph L. (2020). Prognosis of patients with sickle cell disease and COVID-19: A French experience. Lancet Haematol..

[17] Pleasants S. (2014). Epidemiology: A moving target. Nature.

[18] Rees D.C., Williams T.N., Gladwin M.T. (2010). Sickle-cell disease. Lancet.

[19] Tanabe P., Spratling R., Smith D., Grissom P., Hulihan M. (2019). Understanding the complications of sickle cell disease. Am. J. Nurs..

[20] Clift A.K., Saatci D., Coupland C.A., Dambha-Miller H., Hippisley-Cox J. (2021). Sickle cell disorders and severe COVID-19 outcomes: A cohort study. Ann. Intern. Med..

[21] Hoogenboom W.S., Alamuri T.T., McMahon D.M., Balanchivadze N., Dabak V., Mitchell W.B., Morrone K.B., Manwani D., Duong T.Q. (2022). Clinical outcomes of COVID-19 in patients with sickle cell disease and sickle cell trait: A critical appraisal of the literature. Blood Rev..

[22] Panepinto J.A., Brandow A., Mucalo L., Yusuf F., Singh A., Taylor B., Woods K., Payne A.B., Peacock G., Schieve L.A. (2020). Coronavirus disease among persons with sickle cell disease, United States, March 20–May 21, 2020. Emerg. Infect. Dis..

[23] Martin O.Y., Darbari D.S., Margulies S., Nickel R.S., Leonard A., Speller-Brown B., Martin B., Barber J.R., Webb J., Majumdar S. (2023). Clinical outcomes of children and adolescents with sickle cell disease and COVID-19 infection: A year in review at a metropolitan tertiary pediatric hospital. Front. Med..

[24] Sayad B., Karimi M., Rahimi Z. (2021). Sickle cell disease and COVID-19: Susceptibility and severity. Pediatr. Blood Cancer.

[25] Castonguay M., Dakhallah N., Desroches J., Colaiacovo M.-L., Jimenez-Cortes C., Claveau A.-M., Bérubé S., Hafsaoui A.Y., Souza A., Tibout P. (2022). COVID-19 and sickle cell disease in the province of Quebec, Canada: Outcomes after two years of the pandemic. J. Clin. Med..

[26] Mucalo L., Brandow A.M., Dasgupta M., Mason S.F., Simpson P.M., Singh A., Taylor B.W., Woods K.J., Yusuf F.I., Panepinto J.A. (2021). Comorbidities are risk factors for hospitalization and serious COVID-19 illness in children and adults with sickle cell disease. Blood Adv..

[27] Friedman E., Minniti C., Campbell S., Curtis S. (2022). COVID19 vaccination in adults with sickle cell disease is not associated with increases in rates of pain crisis. Hematology.

[28] Nader E., Romana M., Connes P. (2020). The red blood cell—Inflammation vicious circle in sickle cell disease. Front. Immunol..

[29] Patel R., Kaki M., Potluri V.S., Kahar P., Khanna D. (2022). A comprehensive review of SARS-CoV-2 vaccines: Pfizer, moderna & Johnson & Johnson. Hum. Vaccines Immunother..

[30] Link-Gelles R., Levy M.E., Natarajan K., Reese S.E., Naleway A.L., Grannis S.J., Klein N.P., DeSilva M.B., Ong T.C., Gaglani M. (2023). Estimation of COVID-19 mRNA vaccine effectiveness and COVID-19 illness and severity by vaccination status during Omicron BA. 4 and BA. 5 sublineage periods. JAMA Netw. Open.

[31] Fiolet T., Kherabi Y., MacDonald C.-J., Ghosn J., Peiffer-Smadja N. (2022). Comparing COVID-19 vaccines for their characteristics, efficacy and effectiveness against SARS-CoV-2 and variants of concern: A narrative review. Clin. Microbiol. Infect..

[32] Atyabi S.M.H., Rommasi F., Ramezani M.H., Ghane Ezabadi M.F., Arani M.A., Sadeghi M.H., Ahmed M.M., Rajabi A., Dehghan N., Sohrabi A. (2022). Relationship between blood clots and COVID-19 vaccines: A literature review. Open Life Sci..

[33] Alkindi S., Elsadek R.A., Pathare A.V. (2021). Safety warning for ChAdOx1 nCov-19 vaccine in patients with sickle cell disease. Mediterr. J. Hematol. Infect. Dis..

[34] Lee E.J., Cines D.B., Gernsheimer T., Kessler C., Michel M., Tarantino M.D., Semple J.W., Arnold D.M., Godeau B., Lambert M.P. (2021). Thrombocytopenia following pfizer and moderna SARS-CoV-2 vaccination. Am. J. Hematol..

[35] Tiede A., Sachs U.J., Czwalinna A., Werwitzke S., Bikker R., Krauss J.K., Donnerstag F., Weißenborn K., Höglinger G., Maasoumy B. (2021). Prothrombotic immune thrombocytopenia after COVID-19 vaccination. Blood J. Am. Soc. Hematol..

[36] France E.K., Glanz J., Xu S., Hambidge S., Yamasaki K., Black S.B., Marcy M., Mullooly J.P., Jackson L.A., Nordin J. (2008). Risk of immune thrombocytopenic purpura after measles-mumps-rubella immunization in children. Pediatrics.

[37] Soraci L., Lattanzio F., Soraci G., Gambuzza M.E., Pulvirenti C., Cozza A., Corsonello A., Luciani F., Rezza G. (2022). COVID-19 vaccines: Current and future perspectives. Vaccines.

[38] Badier L., Toledano A., Porel T., Dumond S., Jouglen J., Sailler L., Bagheri H., Moulis G., Lafaurie M. (2021). IgA vasculitis in adult patient following vaccination by ChadOx1 nCoV-19. Autoimmun. Rev..

[39] Salvagno G.L., Henry B.M., Pighi L., De Nitto S., Lippi G. (2022). Serum C reactive protein predicts humoral response after BNT162b2 booster administration. J. Infect..

[40] Stuveling E.M., Hillege H.L., Bakker S.J., Gans R.O., De Jong P.E., De Zeeuw D. (2003). C-reactive protein is associated with renal function abnormalities in a non-diabetic population. Kidney Int..

[41] Khera A., Vega G.L., Das S.R., Ayers C., McGuire D.K., Grundy S.M., de Lemos J.A. (2009). Sex differences in the relationship between C-reactive protein and body fat. J. Clin. Endocrinol. Metab..

[42] La Gualana F., Maiorca F., Marrapodi R., Villani F., Miglionico M., Santini S.A., Pulcinelli F., Gragnani L., Piconese S., Fiorilli M. (2023). Opposite Effects of mRNA-Based and Adenovirus-Vectored SARS-CoV-2 Vaccines on Regulatory T Cells: A Pilot Study. Biomedicines.

[43] Fike A., Hartman J., Redmond C., Williams S.G., Ruiz-Perdomo Y., Chu J., Hasni S., Ward M.M., Katz J.D., Gourh P. (2021). Risk factors for COVID-19 and rheumatic disease flare in a US cohort of Latino patients. Arthritis Rheumatol..

[44] Abualfadl E., Ismail F., Shereef R.R.E., Hassan E., Tharwat S., Mohamed E.F., Abda E.A., Radwan A.R., Fawzy R.M., Moshrif A.H. (2021). Impact of COVID-19 pandemic on rheumatoid arthritis from a Multi-Centre patient-reported ques-tionnaire survey: Influence of gender, rural–urban gap and north–south gradient. Rheumatol. Int..

[45] Felten R., Scherlinger M., Guffroy A., Poindron V., Meyer A., Giannini M., Korganow A.S., Sordet C., Chatelus E., Javier R.M. (2021). Incidence and predictors of COVID-19 and flares in patients with rare autoimmune diseases: A sys-tematic survey and serological study at a national reference center in France. Arthritis Res. Ther..

[46] Saligram S., Williams E.J., Masding M.G. (2012). Raised liver enzymes in newly diagnosed Type 2 diabetes are associated with weight and lipids, but not glycaemic control. Indian J. Endocrinol. Metab..

[47] Fahmy A., MacDonald G., Evans A. (2018). Abnormal liver function tests in inflammatory arthritis: Think beyond the DMARDs. Oxf. Med. Case Rep..

